# 

**Published:** 1868-04-01

**Authors:** 


### *CONTENTS:*

The Recognition of American Surgery Abroad. By F. O. Earle, M.D., Chicago, Ill. .... 219
Selections from Surgical Notes. By Prof. Gunn .... 301
Spongiose .... 302
Philadelphia Correspondence .... 303
Cincinnati Correspondence .... 305
Two Clinical Cases. By Tom O. Edwards, M.D., Lancaster, Ohio .... 306

BOOK NOTICES:
Treatise on the Diseases of the Eye. By Carl Stellwag von Carion, M.D .... 310
A Practical Treatise on the Diseases of Women. By T. Gaillard Thomas, M.D .... 313
Atlas of Venereal Diseases. By A. Cullerier .... 313

EDITORIAL:

Clinical Lectures on Diseases of the Eye .... 314

Philadelphia School of Examinations .... 315

Periodical Headache .... 315

Memorandum .... 315

LOOT .... 318

